# Genebank Management Through Microsatellite Markers: A Case Study in Two Italian Peach Germplasm Collections

**DOI:** 10.3390/plants14142139

**Published:** 2025-07-10

**Authors:** Elisa Vendramin, Cássia da Silva Linge, Daniele Bassi, Sabrina Micali, Giorgiana Chietera, Maria Teresa Dettori, Valeria Aramini, Jessica Giovinazzi, Igor Pacheco, Laura Rossini, Ignazio Verde

**Affiliations:** 1 Research Centre for Olive, Fruit and Citrus Crops (CREA-OFA), Council for Agricultural Research and Economics (CREA), Via di Fioranello, 52, 00134 Roma, Italy; elisa.vendramin@crea.gov.it (E.V.); sabrina.micali@crea.gov.it (S.M.); jessica.giovinazzi@crea.gov.it (J.G.); 2 Department of Agricultural and Environmental Sciences–Production, Landscape, Agroenergy, University of Milan, Via Celoria 2, 20133 Milano, Italy; cassia.dasilva@unimi.it (C.d.S.L.); daniele.bassi@unimi.it (D.B.); igor.pacheco@inta.uchile.cl (I.P.); 3 Parco Tecnologico Padano, Via Einstein, Loc. Cascina Codazza, 26900 Lodi, Italy; giorgiana.chietera@iff.com

**Keywords:** cultivar identification, genetic relationship, genetic resources, *Prunus persica* (L.) Batsch, simple sequence repeats (SSR)

## Abstract

Two germplasm collections, comprising 1026 peach accessions located in Italy, were analyzed with 12 simple sequence repeat (SSR) markers. SSR reactions were performed using the multiplex-ready PCR protocol, and 147 alleles were amplified with an average of 12 alleles per locus. BPPCT001 was the most informative marker displaying the highest discrimination power (0.734). The observed heterozygosity showed an average of 0.45 alleles per locus, lower than expected (0.61). The fixation index (F) values were positive in all loci, with an average of 0.27 alleles per locus, suggesting the presence of endogamy. The DNA fingerprinting data allowed the discrimination of 80.95% of the analyzed accessions. If we exclude known sport mutations, known synonymies, and cultivars with the same pedigree, 161 accessions are mislabeled, with an error rate of 16.56% within or between collections. Population structure analysis revealed three subpopulations: modern peach cultivars, modern nectarine cultivars, and a third group mainly comprising traditional peach cultivars. The results obtained in this work will be useful to efficiently manage Genebank, reducing unwanted redundancy, synonyms and homonyms, mislabeling, and spelling errors, as well as identifying parents in controlled crosses.

## 1. Introduction

The peach, *Prunus persica* (L.) Batsch, is a species belonging to the family of the Rosaceae, Spiraeoideae subfamily [1]. The *Prunus* genus encompasses more than 400 species of trees and shrubs and is divided into six subgenera: (1) *Amygdalus* (almonds and peaches), (2) *Prunus* (plums and apricots), (3) *Cerasus* (cherries), (4) *Lithocerasus* (one of the representatives is *Prunus pumila*—sand cherry), (5) *Padus* (*Prunus padus*—bird cherry), and (6) *Laurocerasus* (*Prunus laurocerasus*—cherry laurel). *Prunus* species are sources of nuts, oil, wood, ornamental plants, and fruits such as apricot (*Prunus armeniaca* L.), cherry (*Prunus avium* L.), plum (*Prunus domestica* L.), and peach (*Prunus persica* L.), which cater to different tastes and demands in the market [2,3,4,5].

Peach is cultivated worldwide for the high commercial value of its fruits, commonly consumed raw or canned, or processed as jam, jelly, and juice, and it is appreciated by consumers for its organoleptic and nutritional properties, providing vitamins, minerals, fiber, and antioxidant compounds for healthy diets. According to data from FAOSTAT (http://faostat.fao.org accessed on 15 April 2025), global peach production in 2023 exceeded 27.08 million tons; 76.33% was produced in Asia, 12.82% in Europe, 6.81% in America, 3.79% in Africa, and 0.23% in Oceania. Italy is the fourth largest producer, with an annual production of 1.03 million tons, after People’s Republic of China with 17.50, Spain with 1.38, and Turkey with 1.07 million tons.

Peach is a diploid self-compatible species and stands out as one of the most genetically well-characterized deciduous trees. Due to its characteristics, such as a small genome (227 Mb) and the absence of recent genome-wide duplications [6], it has been adopted as the model species for the Rosaceae family.

Peach has a long history of domestication, starting in northwest China 4000–5000 years ago [7]. Verde et al. [6] published the high-quality draft genome of peach in 2013 and analyzed *Prunus* diversity by resequencing 12 peach cultivars of Eastern or Occidental origin and three close relatives, highlighting two genetic bottlenecks compatible with the domestication events and the dissemination path of the species.

In the last 40 years, the problem of diversity reduction in cultivated plants has become evident in many countries, and germplasm conservation programs have been established to preserve crop diversity. The entry into force of the International Treaty for Plant Genetic Resources for Food and Agriculture (ITPGRFA) raised awareness and gave important input on this issue worldwide as well as in Italy [8]. At first, local and commercial cultivars and crops’ wild relatives were collected, often resulting in large, unbalanced collections of unknown value. At the beginning of this millennium, the main goal shifted from collecting to conserving, managing, characterizing, and exploiting genetic resources [9]. Fruit tree germplasm collections mainly conserved ex situ and in vivo require high financial inputs such as manpower, natural resources, energy, and chemicals; the USDA has estimated that maintaining each tree costs about USD 75–100 per year (https://www.ars.usda.gov/northeast-area/geneva-ny/plant-genetic-resources-unit-pgru/docs/about-pgru/clonal-propagated-crops/ accessed on 19 April 2025). Cryopreservation would be much less costly (approximately USD 1 per plant annually), but its routine implementation is hampered by lack of technology transfer and protocol validation across laboratories [10].

Germplasm collections are prone to errors such as mislabeling and renaming of landraces during their local diffusion, resulting in synonymies (the same genotype with different names) and homonymies (different genotypes with the same name). The level of duplication and errors within and among collections is known to be high, and the elimination of redundancy and errors is economically substantial [9,11]. Incorporating genetic characterization into Genebank management can solve these common issues and lead to more informed decisions and sustainable solutions.

Emanuelli et al. [12], working with 22 SSRs and 384 SNPs in a collection of *Vitis vinifera*, including interspecific hybrids and *Vitis*-related species, found that approximately half of the collection was composed of putative redundant genotypes. A proportion of the redundancy found in this collection was expected, since clonal selections are not generally distinguished by a few molecular markers, but in other cases, mislabeling due to curation errors was the main reason.

Similar results were observed in apple using SSRs to investigate germplasm collections. A portion of the redundancy found was attributed to the difficulty of identifying clonal variants using a limited number of markers. Van Treunen et al. [13], fingerprinting 695 samples from eight Dutch apple collections, found that the redundancy within the total sample was 32%, with the majority between collections, each collection containing only about 50% of unique accessions. The authors proposed that a network of co-operating collection holders would be less costly for more efficient management. Both Liang et al. [14], working in a single Italian collection, and Lassois et al. [15], in a French one, detected about 34% redundancy. Lower values of duplication were found by Larsen et al. [16] in 448 apple accessions, mainly of Danish origin (23%), and by Pina et al. [17], studying a set of 183 accessions coming from mountainous areas in Northeastern Spain (29%). Recently, in apple, Muranty et al. [15,18] and Denance et al. [19] analyzed a large set of accessions with a set of SSRs and the 20K SNP array and developed the “Malus UNiQue genotype code” to generate unique codes for each apple accession. The same approach was recently applied in the Danish “Pometum” collection [16]. To our knowledge, no such system has been reported so far for peach.

Molecular characterization has been the way of choice to explore the extent of variability in germplasm. In fruit trees, the use of molecular markers for identification purposes and repository management began at the end of the nineties. Nowadays the markers of choice are simple sequence repeats (SSRs) and single nucleotide polymorphisms (SNPs). SNPs are the most abundant markers in the genomes and are ideal for high-resolution genome-wide assessments. Nevertheless, SSRs remain the marker of choice for collection management. This is due to their high polymorphism with multi-allelic patterns and their suitability for use with in-house facilities.

While the first studies were limited to a low number of cultivars, in more recent years, faster molecular protocols and/or automation have allowed the screening of a higher number of samples and markers.

In peach, several studies have been conducted using both SSR and SNP markers. Aranzana et al. [20,21] analyzed a collection of 212 Western accessions with a total of 48 SSRs. These researchers aimed at studying genetic diversity in peach germplasm, solving some cases of homonymies and synonymies. Similarly, Li et al. [22] employed the same set of 48 primers to analyze a collection of 434 Oriental peach accessions. They successfully integrated their dataset with that of Aranzana et al. [21], summing up to a total of 653 accessions. This comprehensive analysis led to the identification of three main subpopulations, namely Oriental, Occidental, and landraces, highlighting the higher diversity (higher heterozygosis) of the Oriental germplasm. The modern Occidental breeding subgroup was further divided into two subpopulations: nectarines and peaches. Other studies, with a limited number of accessions and markers, were conducted using SSRs [23,24,25,26].

Micheletti et al. [27] analyzed 1,540 peach accessions from several repositories around the world, including Western and Oriental germplasm, with the 9k SNP peach array [28]. Using about four thousand informative SNPs, a detailed understanding of the genetic diversity and population structure in peach germplasm was proposed. In particular, the study indicates the presence of three distinct genetic groups (namely Occidental breeding, Occidental traditional, and Oriental), with the structure influenced by both the geographic origin and pedigrees. They confirmed the stratification obtained by Li et al. [22] and the further division of the Occidental breeding group (K = 4) into two distinct subpopulations: peaches and nectarines. More recently, genome-wide association studies (GWAS) and structure analyses were performed, highlighting the presence of three main subpopulations, namely wild, landraces, and improved cultivars, in the peach material [29,30,31,32].

In this work, 1026 peach accessions, of which 376 have never been characterized before, were evaluated using a set of 12 SSR markers. The samples investigated came from two peach collections mostly composed of local and commercial peaches. The one held in Rome is the major Italian peach collection and belonging to the National Fruit Germplasm Collection (NFGC) of the Consiglio per la Ricerca in Agricoltura e l’analisi dell’Economia agraria—Centro Olivicoltura, Frutticoltura e Agrumicoltura (CREA-OFA); the other is located in Imola and results from the public–private program MAS.PES (Breeding Apricot and Peach through Marker-Assisted Selection) [33]. The aim was the assessment of the genetic diversity in the two Italian peach collections and to highlight the value of microsatellite markers to support Genebank management.

## 2. Results and Discussion

### 2.1. Genetic Diversity Analysis and Collection Characterization

All 1026 accessions were subjected to the diversity analysis. Among them, 62 names were represented twice each and 6 three times each, within or between the two collections: the number of accessions bearing a distinct name was 952 (Table S1). After genotyping, all the triplicated accessions and 53 couples out of 62 were found to be identical, thus confirming them as replicates of the same genotype. Only one for each group of replicates was retained for further analysis for a total of 961 samples; the genetic profiles for all the accessions are reported in Table S2. Compared with previous studies that analyzed large peach datasets for genetic diversity [21,22,23,25,26,27], a total of 377 peach accessions, comprising traditional and breeding peaches and nectarines (Table S1), were characterized for the first time in this study, while 495 accessions had been previously genotyped using SNPs markers [27].

European and North American germplasm represented the vast majority (89.9%) of the total accessions with a known geographical origin, accounting for 426 and 325 accessions, respectively. This germplasm included several “traditional varieties”, encompassing landraces and local varieties not developed through formal breeding programs.

**Figure 1:**
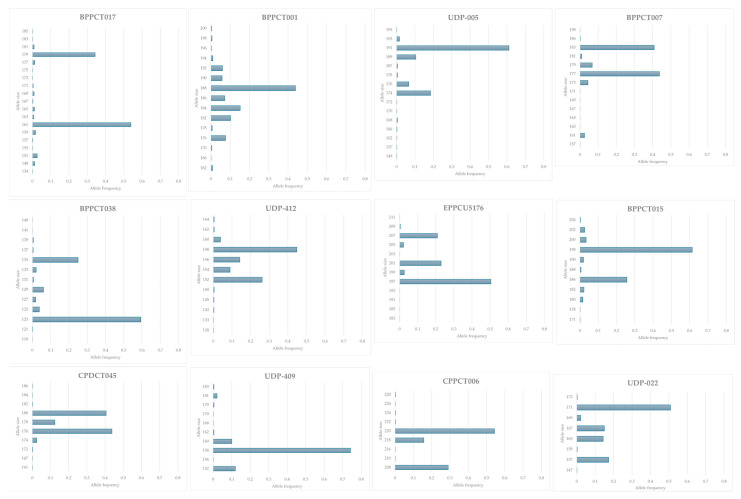
Graphic representation of the allele frequencies for each allele for the 12 SSR markers.

A total of 147 alleles were amplified with 12 SSR loci. The complete list of alleles and their frequency is reported in Figure 1 with an average of the alleles (Table 1). The average number of alleles is 12 per locus, higher than those observed in other works: almost double that in Aranzana et al. [21] and Bouhadida et al. [24], and triple that in Paula et al. [34], but similar to Li et al. [22], Chavez et al. [25], and Shen et al. [26]. The allele number ranged from a minimum of 8 (UDP-022) to a maximum of 19 (for BPPCT017). The observed heterozygosity varied between 0.32 and 0.61 (UDP-409 and EPPCU5176, respectively) with an average of 0.45 per locus. This value was lower than the expected heterozygosity in all loci, with the exception of EPPCU5176, which is the only marker used in this paper deriving from expressed sequence tags (ESTs). A high proportion (72%) of the total were rare alleles (frequency <0.05%) and the allele distributions deviated from Hardy–Weinberg equilibrium (HWE, Table 2). A deviation from HWE with an excess of homozygotes was reported by many authors [20,25,34,35]. This observation is consistent with the occurrence of inbreeding, which is expected in an autogamous species such as peach.

According to Botstein et al. [36], informativeness is limited for loci showing PIC values lower than 0.50. In this study, PIC values were equal or above 0.50 for all loci, except UDP-409. Similar to Bouhadida et al. [24], the most informative marker was BPPCT001 with a PIC value of 0.73, while the least informative was UDP-409 (PIC value of 0.40). The discrimination power (PD) showed an average of 0.79. In line with previous studies [24,37], the marker BPPCT001 showed the highest discrimination power (PD = 0.92). The inbreeding coefficients were positive for all loci, showing an average of 0.27, suggesting the presence of endogamy. A total of 107 rare alleles (MAF < 0.05; Table 2) and 18 private alleles were detected (Table 2). The peach × plum rootstock ‘Citation’ and the peach × *P. webbii* selection IF817023 carry three private alleles each, while 12 accessions, including a peach × almond hybrid (AB/6) and 4 oriental genotypes (‘Chui Huang Tao’, Ferganensis, ‘Shan Dong’, ‘Xiaguang’), carry one each.

Nine couples of accessions sharing the name were shown to be different and were renamed by adding a letter code (‘Azurite’, ‘Harvester’, ‘Limonini’, ‘Lizbeth’, ‘Norman’, ‘Poppa di Venere’, ‘Romagna Red’, ‘Ross’, and ‘Xia Guang’—Table S1), thus reducing the total number of samples with unique labels to 961. In total, 39 couples were replicated between collections, the remaining 24 were duplications inside a single collection.

The dendrogram (Figure S1) representing the similarity among accessions detected 93 groups containing from 2 to 10 accessions for a total of 223 undistinguished genotypes. An accession belonging to *P. davidiana*, an outcrossing wild species close to peach but whose variability was not modified by human selection, was inserted in the analyses as an outgroup.

It stands out that most nectarines (71%) are grouped in a single cluster (Node 9). However, there are a few peaches (26) scattered among them. The remaining nectarines are dispersed among the peaches, individually or in small groups. Consistent with multiple independent origins of the yellow flesh trait [38,39] no clear pattern was detected for flesh color. The only exception was a group of peaches at the top of the dendrogram (Node 10), which primarily consisted of white-fleshed cultivars. A subgroup containing about half of them comes from Asia (Node 10.b) and is a result of breeding programs, often involving American founders, such as Beijing 3 (Elberta) and ‘Hakuto’ and its descendants. ‘Hakuto’ is a Japanese cultivar dating to about 1900 and is possibly a ‘Chinese Cling’ mutant, but Li [22] classifies it as coming from breeding, with structure analysis placing it in the ‘Hakuho’ population. Most of the other samples are European landraces, which, as such, could well have an Asian or old American origin, or groups of modern cultivars with landraces in the pedigree, such as the Cesarini cluster. All flat peaches not coming from modern breeding (9 out of 17) are clustered in Sub-cluster 10.a, including the traditional Sicilian flat peaches and a Chinese one (‘Shao Hong Pantao’). Similarly, Micheletti et al. [27], analyzing the same Chinese flat cultivar together with traditional Sicilian (‘Tabacchiera’) and Spanish flat peaches (‘Paraguayo’), grouped them all in the same cluster together with other Chinese flat peaches and in the Oriental subpopulation, suggesting a common origin of these varietal groups related to the Oriental one. Both the Spanish and Sicilian groups have been cultivated since the XIX century, likely derived from the same source from China. Six flat cultivars, coming from modern breeding, are also grouped together in Cluster 8. Aranzana and coworkers [20,21] found a clear pattern based on flesh melting/non-melting character: both peaches and nectarines were grouped according to fruit flesh texture with only six exceptions. In our dendrogram, a pattern emerges only for the melting flesh type, while the non-melting types are scattered individually or in small groups, often according to their pedigree. Cluster 11 contains mainly melting peaches (279), with the exception of 13 non-melting nectarines and 5 non-melting peaches.

The small group closer to *P. davidiana* (Cluster 1), contains three accessions all coming from China: ‘Pesco Fiore Rosa’ (in English: ‘Pink Flower Peach’) and a sub-cluster (‘Yu Bai’ and ‘Chui Huang Tao’). ‘Pesco Fiore Rosa’ was collected in China (unknown origins) and is an ornamental peach, showing double light pink flowers. ‘Yu Bai’ was obtained in China by crossing, and one of its parents is a landrace according to Cao [40]. The last one is ‘Chui Huang Tao’ (unknown origin).

At a short distance follows a single branch with a selection obtained from CREA-OFA Rome (IF817023), a putative open pollination hybrid of *P. webbii*, a Mediterranean wild almond maintained at CNGF, with an accession of *P. persica*. The selection has small leaves and short internodes. The next cluster (Cluster 3) contains four apparently heterogeneous accessions. The Chinese peach Ferganensis, formerly classified as a distinct species [6], shares a sub-cluster with an old local Italian peach belonging to a varietal population (Poppa di Venere). The other sub-cluster contains ‘Nettarina Pendula’ and ‘Souvenir Nikitsky’, two nectarines of uncertain origin; the first is an ornamental tree with weeping habit, the latter is a tree with no evident ornamental features coming from the Nikita botanical garden in Crimea. This institution actively collected ornamental and edible *Prunus persica* specimens coming from China and from other gardens of the former USSR [3,41]. There are only five selections known to be cultivated as ornamental in this analysis, the one in Cluster 1 (Pesco Fiore Rosa); ‘Nettarina Pendula’ in Cluster 3; ‘Flordahome’, which is also a relative of *P. davidiana*; and ‘Double Crimson’ and ‘Zansetsushidare’, which are at a short distance (Branch 5 and Cluster 6). In Cluster 6 are grouped many accessions carrying ornamental characters, such as ‘S2678’, ‘NJ Weeping’, ‘Zansetsushidare’ (weeping habit, white double flower), and ‘Double Crimson’ (crimson double flower). According to Cao and coworkers [40], the ornamental peaches originated in China from an ancient edible group. This happened about 2000 years after the domestication event which separated peach from its wild progenitor, well before the second bottleneck related to the introduction of peach from Europe to the United States. This suggests that Clusters 3–4–5–6 contain the more diverse accessions of Oriental origin among those sampled in this study. This is also supported by some accessions carrying resistance to diseases: for example, in Cluster 3, ‘Ferganensis’ carries resistance to *Sphaerotheca pannosa* [42], S2678 to green aphids, and ‘Rancho Resistant’ and ‘Higama’ in Cluster 6 to nematodes. Clusters 4 and 6 also contain three out of the four peach × almond hybrids.

The next cluster (Cluster 7, 27 accessions) is also composed by a great majority of peaches, which have in common an origin from warm-climate regions (Brazil, South Italy, Florida, Mexico, Texas) and low chilling requirements.

Based on the molecular study, 227 accessions cannot be distinguished (Table S3). The probability of identity, which is the likelihood that two unrelated individuals by chance share the same multilocus genotype, is low and ranges from 2.98 × 10^−20^ to 3.79 × 10^−5^. In total, 29 accessions refer to known mutations (such as ‘Armking’ and its known sport mutation ‘Armking Precoce’, ‘Escaline’, ‘Silver King’, and ‘Maybelle’), 17 refer to known synonymies (as for ‘Crasiommolo Rosso’ and ‘Graziommolo’), 22 have a shared parent (like ‘Early May’, ‘May Diamond’, and ‘Summer Beaut’) while the remaining 161 refer to accessions that should be different, which thus have to be considered as mislabeling errors. The estimated error rate present in the two collections is equal to 16.56% considering both the accessions (nine) with the same name and different genetic profiles and the genotypes with different labels and the same genetic fingerprint.

Using the 12 SSR markers from the present work, it was possible to distinguish closely related accessions sharing the same parents such as ‘Stark Redgold’ and ‘Maria Aurelia’ (derived from selfing of ‘Stark Redgold’); the couples ‘Orion’ and ‘Venus’ (‘Stark Redgold’ × ‘Flamekist’) and ‘Caldesi 2000’ and ‘Caldesi 2010’ (‘Stark Redgold’ × ‘Snow Queen’); and the three flat peaches ‘Ufo 1’, ‘Ufo 2’, and ‘Ufo 4’ (‘Maybelle’ × ‘Stark Saturn’). ‘Sweet Lady’ (derived from open pollination of ‘Stark Redgold’), ‘Borgia’ (open pollination of ‘Maycrest’), and ‘Capucci 18’ (open pollination of ‘S. Anna Balducci’) showed different fingerprints from their parents and ‘Villa_Ada’, ‘Villa_Doria’, ‘Villa_Giulia’, ‘Villa Adriana’, and ‘Romea’ (all derived from open-pollinated ‘Catherina’) with genetic profiles that are different from their parent and among them (Figure S1). Different genetic patterns were observed between the accessions ‘Loadel’ and ‘Loadel Mutata’. In contrast to two previous studies [21,27], ‘Spring Lady’ and ‘Queen Crest’ showed different genotypes, highlighting a putative mislabeling error in the NFCG collection. However, considering both collections, some accessions could not be distinguished (Figure S1), probably because they are highly genetically related. ‘Bella di Cesena’ and ‘Bella di Cesena Precoce’ showed the same profile for all the markers analyzed, as well as ‘Maycrest’ and ‘Spring Lady’, both known to be sport mutations of ‘Springcrest’ [27]. This was expected for sports that are identical at all loci except for a few mutations [27,43].

The cultivar ‘Queen Crest’ showed a genetic profile identical to ‘Springbelle’. Although pedigree information is not available for ‘Springbelle’, this result is confirmed in two different genetic diversity studies using SSR markers and SNP markers [20,27].

‘Betty’ and ‘Lamone’ were not discriminated, although they stem from different pedigrees: Redwing’ × ‘W6-120’ and ‘Babygold 6’ × ‘Shasta’, respectively. A similar situation was observed with the accessions ‘Appia’ and ‘Brighton’: ‘Appia’ is an open pollination of ‘Southland’ × ‘Pesco Noce 1’, while ‘Brighton’ was selected from the progeny of ‘Sunhigh’ × ‘Redhaven’. Similarly, ‘Ambergold’ and ‘Morsiani 51’ could not be distinguished. Inconsistencies between pedigree information and genetic profiles were also observed in other studies [20,27], and may be explained by labeling errors.

According to pedigree information, the accessions ‘Vivian’ and ‘Fortuna’ have ‘Leader’ as a common parent [44], which could justify the same genetic profile and indicate that a higher number of (or different) markers may be necessary to distinguish accessions sharing a common parentage. Other accessions like ‘Armking Precoce’ and ‘Escaline’ could not be distinguished, probably because both are derived from ‘Armking’ mutations.

In the collections from MAS.PES and NCFG-CREA, 38 out of the 72 accessions in duplicate showed the same profile, confirming their identity. One of the duplications of the accessions ‘Babygold 9’, ‘Flaminia’, ‘Palazzina’, ‘Alitop’, ‘Big Top’, ‘Pieri 81’, ‘Royal Time’, ‘Angelo Marzocchela’, ‘Bella di Cesena’, ‘Botto’, ‘Glohaven’, ‘Grenat’, ‘Magique’, ‘Maria Bianca’, ‘Merril Gem Free’, ‘Nectagrand 1’, ‘Oro A’, ‘Pillar’, ‘Redhaven’; ‘Romagna Bright’, ‘Romagna Gold’, ‘Romagna Red’, ‘Romagna Star’, ‘Romagna Top’, ‘Rosa Dardi, ‘Splendor’, ‘Summer Rich’, ‘Turquoise’, and ‘Zee Glo’ showed missing data for at least one marker. However, the same fingerprint profile was observed in the markers with no missing data. In both collections, the confirmation of their identity can be very useful for tracing plant material and for clarifying and/or confirming the possible parentage of the accessions.

### 2.2. Parentage Analysis

Overall, 53.0% of the comparisons confirm the full matching of declared parentage varying from a minimum of 34% (two known parents) to a maximum of 71.2% (one known parent) (Table 3). This could be ascribed to the controlled crossing method that consists only of the emasculation of the flowers, which does not prevent the access of the bees and unwanted pollination. The parentage analysis of 42 accessions declared to be open-pollinated revealed that 57% could be a result of self-pollination. On the other hand, out of 14 declared self-pollinations checked, only 5 (35.7%) matched. Those numbers are susceptible to slight change if taking possible null alleles into consideration.

When it came to accessions declared as mutants, we observed that 55.2% were matching. This figure does not include accessions with a name suggesting a sport origin, but that, to our best knowledge, have never been described in the literature

In particular, the SSR profile of ‘Gage Elberta’ is identical to that of ‘Elberta’, thus confirming Okie’s hypothesis of ‘Gage Elberta’ being a mutant of ‘Elberta’ [44].

Aranzana et al. [20] analyzed 38 declared pedigrees and found that 100% of the SSR data from open-pollinated seedlings matched their known parent, but this decreased to 47.8 with genotypes with both known parents. This is almost half of the matches, similar to what we observed in our study on a larger sample.

### 2.3. Population Structure

**Figure 2:**
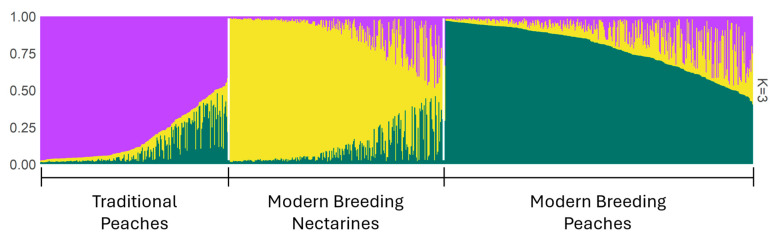
Population structure estimated using the structure at k = 3. The ancestry proportions of the three inferred subgroups are represented by different colors: traditional peaches in purple, modern breeding nectarines in yellow, and modern breeding peaches in dark green.

According to the Evanno method, the population structure analysis revealed three subpopulations (K  =  3) (Figure 2). Considering the membership coefficient Q  ≥  0.80, 477 samples were clustered into three main subpopulations (Table S4). Subpopulation Q2 was mainly composed of nectarines (143 out of 153 accessions, Supplementary Table S4). The other two subpopulations, Q1 and Q3 (134 and 190 accessions, Table S4), were largely composed of fuzzy peaches (301 out of 324 accessions). The remaining accessions (350) could not be assigned under the 80% membership coefficient criterion to any of the three subgroups and remained admixed. The primary reason for this separation observed between the peach (Q1, Q3) and nectarine (Q2) populations lies in their distinct breeding history until recent decades. Breeding programs for nectarines and peaches were largely kept separate, which likely led to different allele frequencies between the two groups. Indeed, the breeding of modern Western nectarines can be traced back to three main cultivars: ‘Quetta’, a local Pakistani cultivar, introduced to Europe and the USA at the beginning of the last century, and ‘Goldmine’ and ‘Lippiat’, both discovered in New Zealand in the early XX century. The origin of the nectarine trait/gene (namely a retrotransposon insertion disrupting the function of the causal gene PpeMYB025) is common to all the Western nectarines known so far [45]. In accordance with our results, Micheletti et al. [27] and Li et al. [22] divided modern Western cultivars into peaches and nectarines.

Subpopulations Q1 and Q3 mainly comprised fuzzy peaches. The biggest group, Q3, is predominantly composed of cultivars obtained during the modern breeding activity in Western countries (129 accessions). This started in the middle of XIX century in the US and can be traced back to a limited number of accessions as founders (‘Chinese Cling’, imported from China in the middle of XIX century, and a few other European cultivars brought to the US by the early settlers [21,22,46]. Most breeding programs in the US and Europe (and partly in Japan and Korea) in the last century rely on this reduced number of genotypes, explaining the difference in allele frequencies observed in this group. The other subgroup, Q1, is equally composed of traditional Western cultivars (56) and modern breeding cultivars (56). The results of this study are in agreement with those obtained by Micheletti et al. [27] that described three main groups, namely Occidental varieties from breeding programs, Occidental landraces, and Oriental accessions. In our study, the number of Oriental accessions was limited (40), thus preventing the detection of a separate group. Most of the cultivars (16 out of 31) obtained by modern breeding activities in South America (Argentina, Brazil, and Mexico) were included in the third group (traditional Western varieties), highlighting the possible origin of these cultivars from a gene pool related to the traditional Western one.

## 3. Materials and Methods

### 3.1. Plant Materials and DNA Extraction

A panel of 1025 peaches (*P. persica*) and a *P. davidiana* accession (a species closely related to *P. persica*) was analyzed. The samples came from two collections: 872 from the NFGC held at CREA-OFA in Rome (41°47′42″ N, 12°33′46″ E) and 154 from the MAS.PES collection in Imola (44°20′12″ N, 11°45′26″ E). The accessions are listed in Supplementary Table S1. The geographical origin (22 countries representing all the continents), the pedigree, and the most important fruit traits evaluated in the fields and/or retrieved from the literature [44,47], are also reported when available.

DNA was extracted from young leaves, using the DNeasy Plant Mini Kit (Qiagen, Germantown, MD, USA) according to the manufacturer’s instructions. Quant-iTPicogreen (Invitrogen Waltham, MA, USA) was used for DNA quantification, and the DNA concentration of each sample was estimated on the basis of a standard concentration curve. The final concentrations of all DNA samples were adjusted to 20 ng/µL.

### 3.2. SSR Analysis

DNA samples were genotyped by 12 SSR markers (Table 1). Markers were selected on the basis of an earlier study by Jouy et al. [48], whose objective was to choose a set of markers for the characterization of large peach collections. SSR reactions were performed using the multiplex-ready PCR protocol [49] with some modifications by Eduardo et al. [50]. After amplification, PCR products, labeled with different dyes, were diluted with 10 μL of distilled water and pooled in a 1:1:1:1 proportion for capillary electrophoresis on an ABI Prism 3730 DNA Analyzer (Applied Biosystems, Waltham, MA, USA). The loading mix consisted of 2 μL of the PCR pool, 10 μL of HI-DI formamide, and 0.15 μL of the GeneScan500 LIZ-250 size standard (ThermoFisher, Waltham, MA, USA).

Allele size was determined with the Gene-Marker demo version 1.70 (SoftGenetics, State College, PA, USA). Using the multiplex-ready approach with “tagged” SSR primers, the expected size of the alleles corresponded to 30 base pairs more than the reported allele sizes [51]. To improve accuracy and allele scoring reliability, two independent readings of the microsatellite sample profiles were made.

### 3.3. Genetic Diversity Analysis

NTSYSpc version 2.11Q [52] was employed to estimate and graphically represent the genetic similarity among cultivars. The SimGend procedure with the Lynch shared band similarity index [53] was used to calculate the similarity; cultivars were then clustered using the SAHN procedure with the unweighted pair-group method (UPGMA).

The number of alleles per locus (Na), the effective number of alleles per locus (Ne), the number of rare (frequency < 0.05) and unique alleles (specific to a genotype), the observed and expected heterozygosity (Ho and He), the polymorphic information content (PIC), the probability of identity (PI), and the combined non-exclusion probability were estimated by the software packages Cervus 3.0.7 [54] and GenAlEx 6.5.0.3 [55]. The discrimination power (DP) of each marker was calculated from the probability of identity (PI) via the following formula: PD = 1 − PI.

### 3.4. Population Structure Analysis

Population structure analysis was conducted with the software Structure 2.3.4 [56], based on Bayesian statistics using 12 SSR markers. The admixture model of ancestry and correlated allele frequencies was adopted to analyze the dataset, with no preliminary subpopulation information. The proportion of the ancestry of each individual was tested considering a K number from 1 to 10, with 10 iterations for each value of K. The settings for burning in and MCMC (Markov Chain Monte Carlo) were 100,000 and 500,000, respectively. To determine the K number, the model established by Evanno was adopted using the structure selector website [57]. CLUMPP v 1.1.2 [58] was used to find the optimal alignment of the 10 independent replications and compute the Q-matrix. The R package ‘pophelper’ v. 2.3.1 [59] was used to graphically display the population structure.

## 4. Conclusions

The molecular genetic characterization of 1026 peach accessions was carried out in order to estimate the genetic diversity and to build a fingerprint database of the germplasm from the MAS.PES and NGFC CREA collections. In accordance with previous studies, the marker with the highest value for the effective number of alleles, PIC, and discrimination power was ‘BPPCT001’. A prevalence of homozygosity was observed for all loci of the evaluated accessions, as expected in the case of an autogamous species. The chosen markers also allowed us to confirm the identity of most duplicated peach accessions in both collections. Some accessions, in a few cases, even if not genetically related, showed the same fingerprinting pattern, and additional SSRs markers are needed to distinguish them. The cultivars analyzed have mostly Occidental origin and represent both traditional and modern germplasm. The present study reports novel fingerprinting and genetic diversity information for 377 peach accessions, while allowing comparisons with the cited previous studies. The genetic analyses, conducted on the 377 accessions, can be useful to breeders to identify material for genetic improvement programs, as well as to gene bank curators to make informed decisions on materials to introduce and maintain. In our case, this work has identified redundancies and errors within and between the two analyzed collections, as well as in the 377 accessions genotyped for the first time in this work, allowing us to upgrade our collections to a better-curated level.

The population structure analysis showed three clusters, with two main subpopulations matching the major varietal groups in pubescent and glabrous peaches. Among the pubescent group, two main groups were identified: one included genotypes obtained from breeding activities and one included most of the traditional landraces. Considering the high values of PIC and PD, the SSR markers used in this work proved to be very efficient for estimating the genetic diversity present in both collections.

The main difference between SNPs and SSRs is their rate of polymorphism. SSR markers are multi-allelic with more than 10 alleles at a single locus (up to 19 in this study), making them useful for fingerprinting and diversity studies, while SNPs are bi-allelic and the most abundant class of markers in the genome. The high-throughput low-cost technologies and their abundance make SNPs the markers of choice for whole-genome analyses such as GWAS. On the other hand, SSR analysis is more flexible and can be carried out by small laboratories.

From a Genebank perspective, SSRs are more suitable and affordable for efficiently managing germplasm collections, reducing unwanted redundancy, mislabeling, and spelling errors.

## Figures and Tables

**Table 1:** Genetic diversity of 961 peach accessions analyzed by 12 SSRs markers. Locus name, number of alleles per locus (Na), effective number of alleles per locus (Ne), information index (I), observed heterozygosity (Ho), expected heterozygosity (He), fixation index (F), polymorphic information content (PIC), discrimination power (PD), and number of rare alleles (Nra) are shown.

Locus	N	Na	Ne	I	Ho	He	F	PIC	PD	Nra
BPPCT001	885	15	4.1	1.81	0.5	0.76	0.34	0.73	0.92	8
BPPCT007	935	14	2.74	1.24	0.5	0.64	0.21	0.57	0.8	11
BPPCT015	871	11	2.24	1.15	0.37	0.55	0.34	0.5	0.75	9
BPPCT017	939	19	2.44	1.23	0.46	0.59	0.22	0.52	0.76	17
BPPCT038	949	13	2.38	1.21	0.44	0.58	0.24	0.53	0.77	10
CPDCT045	909	10	2.69	1.13	0.49	0.63	0.22	0.55	0.78	7
CPPCT006	921	9	2.47	1.03	0.44	0.6	0.26	0.53	0.77	6
EPPCU5176	948	12	2.84	1.24	0.5	0.65	0.23	0.59	0.82	9
UDP-005	841	15	2.36	1.21	0.4	0.58	0.3	0.54	0.78	11
UDP-022	894	8	3.01	1.32	0.42	0.67	0.38	0.63	0.85	4
UDP-409	926	10	1.74	0.88	0.32	0.42	0.24	0.4	0.64	7
UDP-412	932	12	3.35	1.45	0.53	0.7	0.24	0.66	0.87	8

**Table 2:** Genetic profiles of the accessions bearing private alleles (in bold and shaded in light grey).

Sample/SSR	BPPCT001	BPPCT007	BPPCT015	BPPCT017	BPPCT038	CPDCT045	CPPCT006	EPPCU5176	UDP-005	UDP-022	UDP-409	UDP-412
AB/6	170, 190	169, 177	- -	157, 161	125, 129	178, **184**	220, 224	205, 205	162, 174	167, 167	158, 168	154, 154
Chui_Huang_Tao	182, 182	177, 177	186, 186	151, 151	131, **149**	176, 176	- -	193, 193	157, 157	165, 169	181, 181	152, 152
Citation	162, **166**	**171**, 177	198, 198	161, 161	123, 131	176, 178	220, 226	**185**, 197	191, 191	165, 165	158, 158	152, 160
Dourado	186, 188	161, 177	180, 180	151, 165	123, 127	176, 178	208, 220	197, 207	**172**, 191	165, 167	158, 158	152, 158
Ferganensis	182, 182	183, 183	- -	161, 161	123, 123	178, 178	220, 220	209, 209	193, **195**	165, 165	183, 183	162, 162
Fiorenza	188, 188	177, 183	198, 198	161, **185**	123, 123	180, 180	220, 220	197, 205	191, 191	165, 171	152, 158	158, 160
Glowin_Star	170, 190	179, 179	198, 198	**134**, 169	123, 123	176, 186	220, 224	207, 207	174, 189	165, 165	160, 183	156, 156
IF_817023	162, 162	- -	186, 186	155, 161	123, 123	167, 180	**216**, 228	191, 191	174, 174	- -	183, 183	**128**, **133**
P1/12	186, 186	177, **198**	198, 198	161, 161	123, 123	176, 176	220, 220	197, 207	176, 176	157, 157	158, 158	156, 158
P5/645	182, 188	161, 177	186, 186	177, 179	135, 135	176, 180	208, 208	**203**, 207	189, 191	165, 171	158, 158	158, 158
Queen_Ruby	176, 184	179, 183	198, 200	161, 161	123, 123	178, 180	208, 220	201, **211**	189, 191	165, 171	158, 158	152, 156
Red_Robin	182, 182	**165**, 175	198, 198	149, 179	129, 135	176, 180	208, 208	197, 201	191, 191	165, 171	152, 158	152, 158
Shan_Dong	184, 184	161, 177	198, 198	149, 179	123, **141**	176, 180	- -	197, 199	174, 191	- -	158, 181	152, 158
XIAGUANG_a	186, 186	**167**, 183	198, 198	159, 161	123, 123	176, 176	208, 208	201, 201	174, 176	171, 171	158, 160	- -

**Table 3:** Matching of accessions with known pedigree with their putative parents in different types of pedigree.

Type of Pedigree	N°	N Match	% Match
P1 × P2	47	16	34.0
P × Self	14	5	35.7
P op	59	42	71.2
Clones	29	17	55.2
N° Tot	149	80	53.0

## Data Availability

The original contributions presented in this study are included in the article/Supplementary Materials. Further inquiries can be directed to the corresponding authors.

## Supplementary Materials

The following supporting information can be downloaded at https://www.mdpi.com/article/10.3390/plants14142139/s1, Table S1: List of peach accessions genotyped by the 12 SSR markers, and pedigree if known; Table S2: Fingerprint profiles of 961peach samples from the CREA and MAS.PES collections; Table S3: List of accessions with pairwise exact matches and related probability of identity (pID and pIDsib) as calculated by CERVUS; Table S4: Results of the structure analysis with membership coefficients for each cultivar; Figure S1: Dendrogram of 961 peach accessions from 12 SSR markers. Characters are coded by colors in the diagram presented across the dendrogram: nectarines are represented by yellow and peaches by orange (first column), and yellow flesh by green and white flesh by light pink rectangles (second column).

## Abbreviations

The following abbreviations are used in this manuscript.

NGFC CREA	National Fruit Germplasm Collection
MAS.PES	Breeding Apricot and Peach through Marker-Assisted Selection
ITPGRFA	International Treaty for Plant Genetic Resources for Food and Agriculture
GWAS	Genome-Wide Association Studies
GBS	Genotyping by Sequencing
SSRs	Simple Sequence Repeats
SNPs	Single Nucleotide Polymorphisms
PD	Discrimination Power
Ho	Observed Heterozygosity
He	Expected Heterozygosity
PIC	Polymorphic Information Content
PI	Probability of Identity
MAF	Minor Allele Frequency
