# Supplementary material for: Genebank Management Through Microsatellite Markers: A Case Study in Two Italian Peach Germplasm Collections
Source: Plants (Basel). 2025 Jul 10;14(14):2139. doi: 10.3390/plants14142139 (PMC12300406; doi:10.3390/plants14142139)
Supplement: Supplementary file 1 [file plants-14-02139-s001.zip › Supplementary files Rev/Supplementary F1 Rev.pdf]

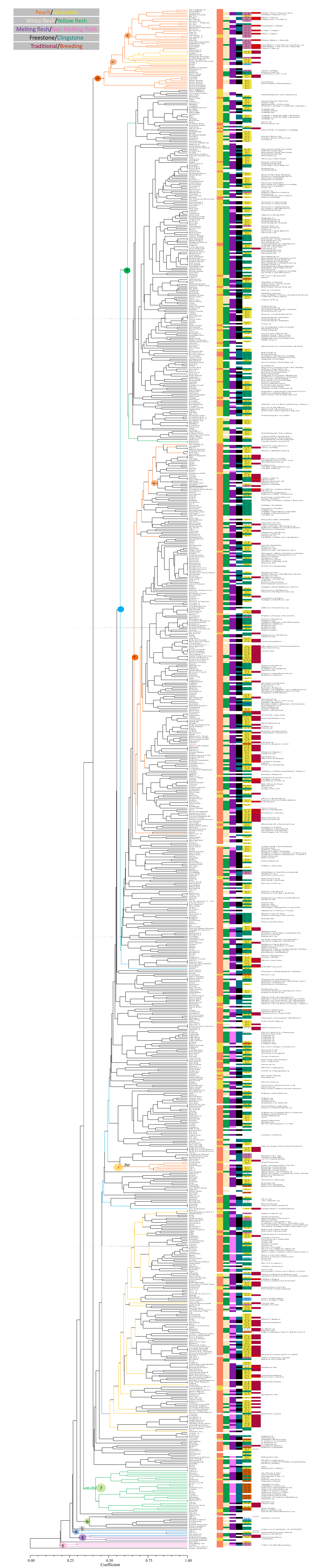

Peach/Nectarine
White flesh/Yellow flesh
Melting flesh/Non Melting flesh
Freestone/Clingstone
Traditional/Breeding
flat
low chill
0.00
0.25
0.50
0.75
1.00
Coefficient
